# Optimization of diagnosis-related groups for patients with acute appendicitis using a machine learning model

**DOI:** 10.3389/fpubh.2025.1581441

**Published:** 2025-09-02

**Authors:** Xinlong Gu, Niannian Li, Heng Wang

**Affiliations:** ^1^Teaching Management Department, The Second Affiliated Hospital of Anhui Medical University, Hefei, China; ^2^Department of Research Administration Office, The First Affiliated Hospital of Anhui Medical University, Hefei, China; ^3^Department of Dean’s Office, The First Affiliated Hospital of Anhui Medical University, Hefei, China; ^4^Department of Health Services Management, School of Health Services Management, Anhui Medical University, Hefei, China

**Keywords:** diagnosis-related groups, acute appendicitis, classification and regression tree, hospitalization cost, machine learning model

## Abstract

**Background:**

The diagnosis-related groups prospective payment system (DRG-PPS) is widely implemented worldwide. Its core components include disease classification and pricing mechanisms. Developing a disease grouping and pricing approach that aligns with local conditions is essential. This study examines the factors influencing hospitalization costs for acute appendicitis (AA) patients and proposes strategies for disease grouping and pricing.

**Methods:**

Stratified random sampling was used to select research sites from provincial, municipal, and county hospitals in Hefei, China. Data were obtained from the hospitalization information systems of three hospitals from 2017 to 2019. The primary diagnosis was defined as AA. Single-factor analysis and multiple linear stepwise regression were used to identify the main factors influencing hospitalization costs. Additionally, a classification and regression tree (CART) model, based on the exhaustive chi-square automatic interaction detection (E-CHAID) algorithm, was applied to establish the DRG grouping model.

**Results:**

A total of 4,066 patients were included. Significant differences in hospitalization costs were observed based on length of stay (LOS), marital status, surgery, and hospital level (*p* < 0.05). By incorporating age, type of surgery, and LOS into the CART model, AA inpatients were classified into 10 DRG groups. The standardized disease cost ranged from 3,047 CNY to 15,569 CNY.

**Conclusion:**

Hospitalization costs for AA patients are primarily influenced by LOS, marital status, surgery, and hospital level. The decision tree model provides a basis for DRG grouping. Health administration departments may consider implementing precise and individualized hospitalization cost reimbursement mechanisms accordingly.

## 1 Introduction

Appendicitis is an inflammation of the vermiform appendix (1) and is among the most common surgical emergencies in both children and adults (2). Globally, the annual incidence of appendicitis ranges from 96.5 to 100 cases per 100,000 individuals (1). Acute appendicitis (AA), a prevalent form of appendicitis, is most frequently diagnosed between the ages of 10 and 30, with the lowest incidence occurring in children aged nine years or younger (3). The typical symptoms of appendicitis include vague periumbilical pain, anorexia, intermittent vomiting, nausea, pain radiating to the lower right abdomen, and low-grade fever (4). When symptoms persist for more than 24 h, the risk of localized ischemia, perforation, gangrene, and abscess formation increases. As a result, AA complications pose a significant threat to public health.

The clinical diagnosis of AA is based on patient history, physical examination, laboratory findings, and imaging studies (5). Prompt treatment is essential to prevent severe complications. Open and laparoscopic appendectomy are the primary treatment methods, and antibiotic therapy has been shown to be a viable and effective option for acute uncomplicated appendicitis (6). The Society of American Gastrointestinal and Endoscopic Surgeons (SAGES) recommends laparoscopic appendectomy as the first-line treatment for adult patients with acute uncomplicated appendicitis (7, 8). However, with increasing age, both recovery time and length of stay (LOS) following AA surgery tend to be longer. Additionally, the mortality rate for AA rises significantly in individuals aged 65 and older (3). The high incidence and mortality rates, coupled with the substantial treatment costs, have created a severe clinical and economic burden globally (8, 9). Globally, the economic burden of AA is substantial. For instance, the average cost of hospitalization for appendicitis in the United States is approximately $13,000 per case, with the costs rising significantly for complicated cases such as perforated appendicitis (9). In China, the average hospitalization cost for acute appendicitis varies by region but typically ranges between 5,000 to 15,000 RMB per patient, depending on the complexity of the case and the type of surgical intervention required (10). These costs reflect not only the price of surgery and hospital stay but also the long-term healthcare expenses related to complications, extended recovery times, and follow-up care. The growing financial burden associated with AA, alongside its high incidence and mortality rates, highlights the need for more efficient cost-control measures, including the implementation of DRG systems. Reducing hospitalization and treatment costs through optimized healthcare management can mitigate the economic strain on both patients and healthcare systems. To reduce the financial strain on patients, government interventions aimed at controlling medical expenses are necessary.

Diagnosis-related groups (DRG) are widely recognized as one of the most advanced medical payment management systems. Numerous studies have demonstrated its effectiveness in controlling medical costs and reducing the financial burden on patients (10). Initially developed at Yale University, DRG was first implemented in the United States in 1983 (11). It is a payment model that classifies diseases with similar clinical symptoms and resource consumption into specific groups. These classifications serve as the foundation for medical institutions to generate patient bills and for insurance agencies to establish reimbursement standards (12). However, DRG grouping rules vary across different countries and regions.

The DRG system was introduced in China in 1994, with its feasibility first studied by Huang (13). Due to economic disparities between regions, the Chinese government allows each locality to develop grouping rules that reflect its specific conditions. Over time, various DRG models such as BJ-DRG, C-DRG, and CN-DRG have been established in China (14). Significant differences exist in the design of DRG grouping rules across different regions. To standardize grouping criteria in pilot cities, the National Healthcare Security Administration (NHSA) launched the China Health and Safety Diagnosis-Related Group (CHS-DRG) in 2019. As a result, well-structured DRG grouping is essential for an effective cost-payment system, helping to regulate medical expenses and monitor unreasonable charges.

Recently, machine learning models have gained significant attention for their potential to enhance the accuracy and efficiency of DRG grouping systems. Several studies have utilized machine learning techniques to refine disease classification and predict healthcare costs, particularly for specific conditions. For instance, algorithms have been used to forecast hospitalization costs, length of stay, and patient outcomes in diseases, such as heart failure, diabetes, and cancer (15, 16). These studies have demonstrated promising results in optimizing DRG systems by improving cost prediction accuracy and resource allocation. However, the application of machine learning to DRG models for acute appendicitis remains largely unexplored, with limited research examining its potential benefits. A review of existing machine learning-based DRG models for diseases with similar clinical characteristics could help contextualize this study, providing valuable insights into how such approaches could contribute to better cost control and more effective resource management in the DRG framework. This study analyzed hospitalization data of AA patients in Hefei, China, one of the pilot cities for NHSA grouping. First, univariate analysis was conducted to identify factors influencing hospitalization costs for AA patients. Next, multiple stepwise linear regression analysis was used to select predictive factors for the machine learning model. Finally, based on these predictive factors, a decision tree model was developed to estimate hospitalization costs for AA patients, and a grouping scheme was proposed to align with the local healthcare landscape. This study aims to provide theoretical support for assessing the applicability of the DRG prospective payment system.

## 2 Materials and methods

### 2.1 Study design and data collection

This cross-sectional study employed stratified random sampling to ensure a representative sample. Hospitals in China are categorized into provincial, municipal, and county-level institutions. One hospital from each level was randomly selected. Medical records and cost data for inpatients diagnosed with acute appendicitis (AA) as the primary diagnosis (ICD-10 code K35) from 2017 to 2019 were extracted from the hospital information system. Patient information, including age, gender, type of surgery, insurance type, LOS, and hospitalization costs, was collected. Cases with incomplete data were excluded from the analysis; however, the proportion of missing data for each variable was not specified. The missing data rate was calculated for each variable, and any variable with more than 10% missing values was excluded from the final analysis to prevent bias. For variables with less than 10% missing data, imputation was performed using the multiple imputation method (MICE) to preserve sample size and ensure robust estimates. The imputation process was conducted under the assumption that data were missing at random (MAR). Sensitivity analysis was conducted to assess the impact of imputation on the results, and no significant differences were found between the complete case and imputed datasets. This approach minimized the potential for bias due to missing data and enhanced the validity of the study findings. After excluding cases with incomplete data, a total of 4,066 cases were included.

### 2.2 Statistical analysis

First, the Chi-square test was used to analyze differences between groups. The demographic characteristics of inpatients were summarized as rates and percentages according to hospital levels.

Second, the *t*-test and analysis of variance (ANOVA) were conducted to examine factors influencing hospitalization costs in AA patients. A multiple linear regression model was then established, incorporating statistically significant variables from the univariate analysis as independent factors. Multicollinearity was assessed using the variance inflation factor (VIF) and tolerance. A VIF > 10 or tolerance <0.1 was considered indicative of multicollinearity.

Third, to further explore the interactive relationships between hospitalization costs and demographic or health-related variables, a classification and regression tree (CART) model was employed. This machine learning model is effective in identifying complex interactions among factors that traditional analytical methods may overlook (15). All variables found to be statistically significant in the univariate regression model were included in the CART model. To optimize the classification tree, the exhaustive chi-square automatic interactive detection (E-CHAID) algorithm was used as the growing method. The validity of the grouping was assessed by evaluating heterogeneity between groups and homogeneity within groups based on data distribution. The CART model was selected for this study because of its ability to capture complex interactions between multiple variables and its clear interpretability through decision rules. In contrast to traditional linear models, which assume linear relationships between predictors and outcomes, CART does not require such assumptions and is well-suited for modeling non-linear relationships. This flexibility is particularly useful for healthcare data, where the relationships between factors, such as demographic characteristics, surgical interventions, and hospitalization costs are often non-linear and intricate. While alternative machine learning models, such as Random Forests and Gradient Boosting, were considered, they were ultimately not chosen for this study. These ensemble models typically present improved predictive accuracy, while their “black-box” nature limits their interpretability, which was a key consideration for this study. Since the goal was to generate transparent, interpretable insights into the factors influencing hospitalization costs, the CART model was preferred for its ability to produce easily understandable decision trees. Logistic regression was also evaluated, while was regarded inappropriate for this analysis, as it assumes a binary outcome, whereas hospitalization costs are continuous. Although the CART model effectively captures the relationships in the dataset, future research should compare its performance with other models, such as Random Forests or Gradient Boosting, to assess potential gains in predictive accuracy.

Categorical variables with two levels (e.g., marital status) were entered into the regression model using binary coding. For variables with more than two categories (e.g., hospital level, insurance type), dummy variables were created, with one category designated as the reference group. This approach allowed for the comparison of each category’s effect relative to the reference category while ensuring appropriate model specification. All collected data were entered into Excel 2010 (Microsoft Corporation, Redmond, WA, United States), and statistical analyzes were performed using SPSS 26.0 (SPSS Inc., Chicago, IL, United States). A *p*-value <0.05 was considered statistically significant.

### 2.3 Variables

The dependent variable is the hospitalization costs (Y), and the independent variables are gender (X1, male, female), age (X2, years, <11, 11–20, 21–30, 31–40, 41–50, 51–60, 61–70, >71), marital status (X3, married or cohabited, single), insurance type (X4, urban resident basic medical insurance (URBMI), urban employee basic medical insurance (UEBMI), new rural cooperative medical insurance (NRCMI), other), LOS (X5, days, 1–3, 4–6, 7–9, 10–12, >12), presence of complications (X6, yes, no), whether surgery (X7, yes, no), type of surgery (X8, laparoscopic surgery, laparotomy), and hospital level (X9, provincial hospitals, municipal hospitals, and county hospitals). It should be clarified that the term “hospitalization costs” in this study refers to the total charges billed to patients or their insurers during the hospitalization episode, as recorded in the hospital information system. These figures may not necessarily reflect the exact economic cost incurred by the provider but are used here as proxies for resource consumption in DRG classification.

## 3 Results

### 3.1 Results of descriptive analysis

A total of 4,066 patients were included in the study, with 1,197 patients (29.4%) from provincial hospitals, 1,200 patients (29.5%) from municipal hospitals, and 1,669 patients (41.1%) from county hospitals. The sample consisted of 2,103 male patients (51.7%) and 1,963 female patients (48.3%). Patient ages ranged from 2 to 95 years, with a mean age of 39.51 years. The majority of patients were married or cohabiting (72.8%), selected basic medical insurance (80.3%) (including UEBMI, URBMI, and NRCMI), and had a LOS of 4–9 days (75.6%). Most patients had simple appendicitis without complications or comorbidities (CC) (75.3%), while 1,006 patients presented with CC (including perforation, peritonitis, peripheral abscess, perforation with localized peritonitis, and perforation with diffuse peritonitis). Surgical intervention was required for 73.6% of AA patients, with 78.7% of these cases undergoing laparoscopic appendectomy.

### 3.2 Results of single factor analysis

The *t*-test and analysis of variance (ANOVA) were conducted to perform univariate analysis of hospitalization costs in AA patients. Hospitalization costs increased with age, reaching the highest levels in patients aged >71 years (11,557.56 ± 7,582.89 CNY). Higher costs were also observed among married or cohabiting patients (10,408.92 ± 5,740.97 CNY), those with an LOS > 12 days (18,579.15 ± 11,437.15 CNY), and those who underwent surgery (11,888.28 ± 5,223.94 CNY). Patients using the NRCMI payment method (8,600.93 ± 4,859.97 CNY) and those treated in county-level hospitals (7,583.15 ± 4,031.00 CNY) had lower hospitalization costs. However, no statistically significant correlation was found between gender, CC, type of surgery, and hospitalization costs, as shown in Table 1.

**Table 1:** Single factor analysis of hospitalization costs in acute appendicitis.

Variables	*N*	Hospitalization costs (CNY) (Mean ± SD)	*F*/*T*	*P* value
Gender			0.001	0.979
Male	2,103	10291.10 ± 5523.76		
Female	1963	9959.96 ± 5206.85		
Age			9.917	<0.001
< 11y	177	8727.05 ± 3644.17		
11–20y	549	9030.19 ± 4239.75		
21–30y	892	9948.36 ± 4343.31		
31–40y	617	10217.28 ± 5115.36		
41–50y	623	10162.25 ± 5308.17		
51–60y	493	10743.12 ± 5893.17		
61–70y	392	10593.81 ± 6636.68		
> 71y	323	11557.56 ± 7582.89		
Marital status			37.924	<0.001
Married or cohabited	2,959	10408.92 ± 5740.97		
Single	1,107	9388.97 ± 4156.68		
Insurance type			70.310	<0.001
UEBMI	1,330	11198.92 ± 5299.19		
URBMI	432	11278.92 ± 4223.76		
NRCMI	1,505	8600.93 ± 4859.97		
Other	799	10615.91 ± 6243.75		
LOS			182.389	<0.001
1–3d	598	7930.70 ± 4666.67		
4–6d	1792	9540.04 ± 4018.34		
7–9d	1,279	10303.66 ± 4503.70		
10–12d	230	13365.90 ± 6607.39		
> 12d	167	18579.15 ± 11437.15		
CC			2.842	0.092
Yes	1,006	12613.32 ± 5651.61		
No	3,060	9315.22 ± 5020.52		
Surgery			11.888	0.001
Yes	2,994	11888.28 ± 5223.94		
No	1,072	5223.94 ± 3751.95		
Type of surgery			0.071	0.791
Laparoscopic	2,355	12866.18 ± 4426.90		
Laparotomy	639	8284.28 ± 4079.97		
Level of hospital			377.687	<0.001
Provincial hospital	1,197	11794.48 ± 6005.37		
Municipal hospital	1,200	12016.08 ± 4900.37		
County hospital	1,669	7583.15 ± 4031.00		

*P* < 0.05 was considered statistically significant. URBMI, urban resident basic medical insurance; UEBMI, urban employee basic medical insurance; NRCMI, new rural cooperative medical insurance; CC, comorbidity and complication; LOS, length of stay.

### 3.3 Results of multivariate linear regression analysis

A collinearity diagnostic analysis was conducted before performing multiple linear regression. No collinearity was detected among the variables in this study (Supplementary Table S1). All variables found to be statistically significant in the univariate regression model were included as independent variables in the multiple linear regression model. These variables included age (X2), marital status (X3), insurance type (X4), LOS, (X5), surgery (X7), and hospital level (X9). The results of the multivariate linear regression analysis are shown in Table 2. The multiple linear regression equation is formulated as:


$$\begin{array}{l} Y=13447.64+2642.58X5-4298.61X8-1840.27\mathrm{X9}\\ +389.81X2-476.46\mathrm{X3} \end{array}$$

**Table 2:** Multivariate linear regression analysis of hospitalization costs in acute appendicitis.

Variables	B	*T*	*P* value	95%CI		Collinearity	
				Lower	Upper	Tolerance	VIF
Constant	13447.64	30.67	<0.001	12587.95	14307.33	–	–
LOS	2642.58	34.44	<0.001	2492.13	2793.03	0.885	1.130
Type of surgery	-4298.61	−25.86	<0.001	−4624.61	−3972.61	0.873	1.145
Level of hospital	−1840.27	−21.48	<0.001	−2008.25	−1672.28	0.817	1.225
Age	389.81	8.82	<0.001	303.14	476.48	0.602	1.661
Marital status	−476.46	−2.67	<0.001	−826.79	−126.12	0.615	1.626

*P* < 0.05 was considered statistically significant. LOS, length of stay; B, unstandardized coefficients; VIF, Variance inflation factor; *p* < 0.05 was considered statistically significant.

The model demonstrated statistical significance, with a corrected *R*^2^ = 0.460, *F* = 511.778, and *p* < 0.001. At a significance level of *α* = 0.05, the multiple linear regression equation was confirmed to be statistically significant.

### 3.4 Results of classification and regression tree model

**Figure 1:**
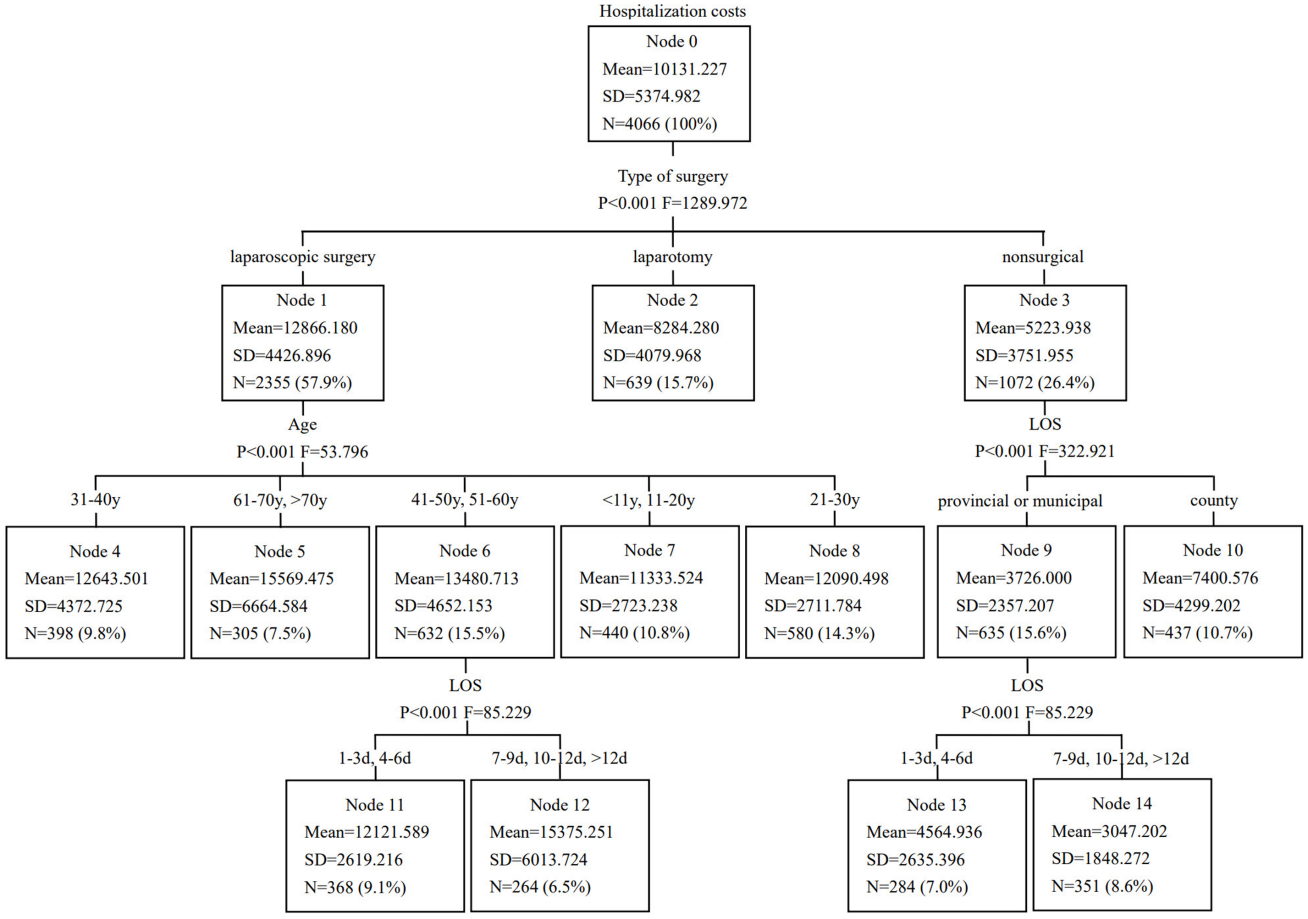
Classification and regression tree model of hospitalization costs in patients with acute appendicitis.

A CART model was developed and pruned using the E-CHAID algorithm. Inpatient hospitalization costs were set as the dependent variable, while LOS, type of surgery, hospital level, and age were used as classification nodes. The model parameters were configured as follows: the maximum number of tree layers was set to 3, the minimum sample size for the parent node was 400, the minimum sample size for the child node was 250, and the significance level for node splitting was *α* = 0.05. The number of DRG groups was determined based on the E-CHAID algorithm’s optimization, which identified 10 distinct groups with significant differences in hospitalization costs. The Kruskal-Wallis rank sum test results indicated that the ten case combinations identified had significantly different hospitalization costs. The CART model results are presented in Figure 1.

Although alternative group numbers (e.g., 5 or 15) were considered, the 10-group configuration emerged as the most statistically significant, as it best captured the variability in hospitalization costs. Table 3 displays the optimized DRG grouping scheme for AA patients. The DRG 1 group had the highest number of cases, accounting for 15.7% of patients, followed by the DRG 3 group, which comprised 14.3%. The model’s validity was assessed based on heterogeneity between groups and homogeneity within groups, with the coefficient of variation (CV) among the ten DRG groups being less than 0.8, indicating a high degree of cost homogeneity in each group. Future studies will further refine the grouping approach by testing alternative configurations (such as 5 or 15 groups) and using model selection criteria, such as AIC, BIC, or cross-validation to compare and justify the optimal number of groups.

**Table 3:** Results of DRG grouping in patients with acute appendicitis.

Groups	Grouping rules	*N* (%)	Mean	SD	CV
DRG 1	Laparotomy	639 (15.7)	8284.28	4079.97	0.49
DRG 2	Laparoscopic surgery, age <20y	440 (10.8)	11333.52	2723.24	0.24
DRG 3	Laparoscopic surgery, age 21-30y	580 (14.3)	12090.50	2711.78	0.22
DRG 4	Laparoscopic surgery, age 31-40y	398 (9.8)	12643.50	4372.43	0.35
DRG 5	Laparoscopic surgery, age >61y	305 (7.5)	15569.48	6664.58	0.43
DRG 6	Laparoscopic surgery, age 41-60y, LOS 1-6d	368 (9.1)	12121.59	2619.22	0.22
DRG 7	Laparoscopic surgery, age 41-60y, LOS > 7d	264 (6.5)	15375.25	6013.72	0.39
DRG 8	LOS 1-6d, provincial or municipal hospital	284 (7.0)	4564.94	2635.40	0.58
DRG 9	LOS 1-6d, county hospital	351 (8.6)	3047.20	1848.27	0.61
DRG 10	LOS > 7d	437 (10.7)	7400.58	4299.20	0.58

LOS, length of stay; SD, standard Deviation; CV, coefficient of variation.

## 4 Discussion

In this study, the majority of AA patients were between 11 and 50 years old, accounting for 78.06% of the cohort. The age range was broad, with the youngest patient being 2 years old and the oldest 95 years old. AA is common across various age groups, a finding consistent with previous research (16). Additionally, county hospitals treated the highest number of cases (1,669 patients, 41.05% of the sample). This study indicated differences in LOS, CC, surgery, and type of surgery among AA patients across different hospital levels. The LOS for most patients ranged from 1 to 9 days, with the majority having simple AA. Surgical intervention is the primary treatment, which aligns with the known characteristics of AA (17, 18). As a common acute condition, AA follows a well-defined treatment pathway, and postoperative recovery is generally favorable following appendectomy (19). This study revealed significant differences among AA patients based on age, marital status, insurance type, LOS, surgical intervention, and hospital level. A negative correlation was identified between hospital level, insurance type, marital status, type of surgery, and hospitalization costs. Patients with the lowest hospitalization costs were those who were single, covered by NRCMI, treated in county hospitals, and underwent open appendectomy. Conversely, age, LOS, CC, surgery, and hospitalization costs showed a positive correlation. Hospitalization costs increased with age, longer LOS, presence of CC, and surgical intervention. A strong association was identified between surgery and the type of surgery in relation to hospitalization costs. However, it is noteworthy to clarify that surgery and the type of surgery are inherently linked variables, and this should not be interpreted as multicollinearity. Rather, this association highlights that different surgical approaches (e.g., laparoscopic vs. open appendectomy) have distinct effects on resource utilization, which may in turn influence hospitalization costs. The correlation can be attributed to both the characteristics of the surgical procedures and the underlying structure of medical payment systems. Extended hospitalization, more complex surgeries, and the presence of comorbidities all contribute to higher resource consumption, ultimately leading to increased costs.

Age was identified as the second-level classification node. As individuals age, physiological functions decline, the prevalence of underlying diseases increases, complications become more frequent, and disease prognosis tends to be slower. Consequently, older patients require more medical resources. Among those aged 60 and above, most have comorbidities that directly contribute to increased resource consumption. In this study, patients over 60 years old had the highest hospitalization costs, a finding consistent with research conducted in Germany and the United States (20, 21). Although age was one of the key splitting variables in the CART model, it was not the sole or primary determinant of DRG grouping; rather, it interacted with other clinically relevant and resource-related factors, such as type of surgery, LOS, and hospital level, supporting a multidimensional rather than age-dominant classification approach for determining provider payments.

The third-level classification node was divided into two factors: hospital level and LOS. Patients receiving treatment at county-level hospitals had the lowest hospitalization costs. Under China’s tiered healthcare system, county hospitals have a higher medical insurance reimbursement ratio than provincial or municipal hospitals. Additionally, patients in county hospitals typically present with less severe conditions and are more likely to receive non-surgical treatments, such as medication or open appendectomy. Among all factors, LOS had the most significant impact on hospitalization costs, aligning with findings from related studies in China (22–24). In the DRG grouping guidelines of developed countries, including the United States, United Kingdom, and Poland, LOS is considered a key factor (25). As widely reported, hospitalization costs increase with longer LOS. This correlation exists because LOS reflects not only medical resource consumption but also disease severity and healthcare efficiency. Therefore, measures should be implemented to reduce LOS, improve bed turnover rates, and lower hospitalization costs. It is important to note that the hospitalization costs analyzed in this study refer to the actual charges recorded in the hospital information system, which may not fully represent the underlying resource-based costs of care. These charges reflect the billed amounts and are subject to regulatory policies, hospital pricing strategies, and insurance reimbursement frameworks. In some cases, charges may be higher or lower than the true economic cost due to subsidies, profit margins, or government-imposed price ceilings. Nonetheless, the use of these data remains valid for DRG optimization, as DRG systems are primarily designed to classify and reimburse cases based on relative resource consumption across homogeneous patient groups, rather than to capture precise economic costs on an individual basis.

The evaluation indicators from the CART model confirm that the DRG grouping scheme for AA in this study is well-structured. Our findings demonstrate significant cost homogeneity within each DRG group and notable cost heterogeneity between groups, aligning with the fundamental principles of DRG classification. While this study proposed a new 10-group DRG classification for AA patients, a comparative analysis with existing DRG systems, such as the CHS-DRG, is warranted. In particular, systems, such as BJ-DRG, CN-DRG, and CHS-DRG typically group AA cases into broader categories based primarily on surgical status and the presence of complications. These existing systems, however, may lack the granularity needed to capture regional variations in hospital practices and resource consumption, which can lead to reduced specificity in cost prediction and reimbursement allocation. In contrast, the proposed 10-group classification provides a more detailed stratification, allowing for more accurate cost predictions by incorporating not only the type of surgery and complications, but also age, LOS, and hospital level. This finer classification may reduce cost heterogeneity within groups, with a CV of less than 0.8, suggesting high-cost homogeneity. The greater stratification aligns with international DRG principles that emphasize balancing complexity and efficiency in grouping to avoid unnecessary fragmentation. Moreover, the CHS-DRG system may not fully reflect the local healthcare context, particularly the differences in hospital resources and practice patterns across varying hospital levels. This study’s approach, by explicitly considering these local factors, may possess advantages in terms of both policy relevance and practical implementation for medical insurance departments in China. The enhanced granularity of our model can support more precise reimbursement schemes and better reflect resource consumption, making it a potential improvement over current DRG systems. While the CHS-DRG system and other national groupers have a broader classification scheme, the optimized model presents a refined alternative that can promote future updates or the development of a more regionally adaptable system. It is noteworthy that in several DRG systems, such as the Australian Refined Diagnosis Related Groups (AR-DRG), the Patient Clinical Complexity Level (PCCL) is used to quantify the overall clinical complexity of a case by integrating the effects of multiple comorbidities and complications (11). The PCCL provides a more granular and standardized measure of resource intensity. However, PCCL values were not available in the dataset used for this study, as the hospital information systems did not record the necessary variables to compute this index. Therefore, we relied on the binary coding of complications and comorbidities (yes/no) as a simplified proxy for patient complexity. While this approach has limitations in capturing nuanced clinical severity, it reflects current data practices in many Chinese hospital systems and provides a pragmatic basis for DRG grouping in this context. Future research should incorporate PCCL or equivalent metrics once the required data infrastructure becomes available. Existing DRG groupers in China (e.g., BJ-DRG, CN-DRG, CHS-DRG) often group AA cases into broad categories based primarily on surgical status and presence of complications (26, 27). However, these systems may lack regional adaptability and granularity needed to reflect local practice patterns and resource variation. Our optimized DRG model introduces a more remarkable stratification, providing improved cost homogeneity and reflecting local hospital-level differences. This evidence-based classification may promote the refinement of current groupers or guide future updates to the national system.

This study presents several advantages. Firstly, the data from Hefei, China, ensure representativeness and minimize regional cost variations. Secondly, machine learning models enable a comprehensive analysis of multiple variables and produce interpretable decision tree diagrams for decision-making. Thirdly, using historical data to estimate standard costs highlights differences in resource consumption, providing valuable insights for future DRG-based payment reforms. While the study did not include a formal clinical severity index, selected predictors, such as age, length of stay, and surgery type serve as practical proxies for disease complexity. The study expands on DRG classifications for AA by creating 10 groups, providing a finer classification for more accurate cost prediction. This can support better reimbursement schemes and help local health authorities design policies that align with resource consumption patterns in AA patients.

However, this study has certain limitations. Firstly, it concentrated on AA patients, and the findings might not be directly applicable to other conditions. Secondly, while inpatient fees were used as a proxy for costs, they might not fully represent the actual economic resources used during treatment. The data, collected from hospitals in Hefei, might also limit the generalizability of the results to other regions. Future research should explore the applicability of this DRG model to other diseases and conduct prospective studies in different settings to validate the model’s broader use.

## 5 Conclusion

This study provides a theoretical basis for DRG grouping of AA, identifying key variables influencing hospitalization costs and utilizing the CART model for case combination classification. Our findings indicate that LOS, hospital level, and type of surgery serve as primary nodes for DRG grouping. Using a machine learning model, patients were classified into ten DRG groups, with significant cost differences observed between groups, while cost variations within groups remained relatively small. The study validates the applicability of disease grouping based on multivariate statistical analysis and machine learning models in AA patients in Hefei, China, demonstrating the feasibility of case combination classification. Furthermore, our findings provide a useful reference for improving disease diagnosis grouping systems in other regions and countries.

## Data Availability

The raw data supporting the conclusions of this article will be made available by the authors, without undue reservation.
