# Supplementary material for: Optimization of diagnosis-related groups for patients with acute appendicitis using a machine learning model
Source: Front Public Health. 2025 Sep 2;13:1581441. doi: 10.3389/fpubh.2025.1581441 (PMC12436493; doi:10.3389/fpubh.2025.1581441)
Supplement: Supplementary file 1 [file Table_1.docx]

**Table S1.** Test results of correlation between variables

| **Variables** | **Level of hospital** | **Insurance type** | **Gender** | **Age** | **Marital status** | **LOS** | **CC** | **Surgery** | **Type of surgery** | **Hospitalization costs** |
| --- | --- | --- | --- | --- | --- | --- | --- | --- | --- | --- |
| Level of hospital | 1 | — | — | — | — | — | — | — | — | — |
| Insurance type | 0.249** | 1 | — | — | — | — | — | — | — | — |
| Gender | -0.036* | -0.033* | 1 | — | — | — | — | — | — | — |
| Age | 0.062** | -0.071* | 0.063** | 1 | — | — | — | — | — | — |
| Marital status | -0.041** | 0.072** | -0.107** | -0.635** | 1 | — | — | — | — | — |
| LOS | 0.267** | 0.046** | -0.022 | 0.162** | -0.062** | 1 | — | — | — | — |
| CC | -0.144** | -0.005 | -0.061** | -0.013 | -0.004 | 0.142** | 1 | — | — | — |
| Surgery | -0.207** | -0.062** | -0.030 | -0.141 | 0.071** | 0.019 | 0.249** | 1 | — | — |
| Type of surgery | 0.350** | 0.106** | 0.013 | 0.044* | -0.018 | 0.188** | -0.094** | — | 1 | — |
| Hospitalization costs | -0.379** | -0.125** | -0.022 | 0.091** | -0.072** | 0.244** | 0.269** | 0.614** | -0.511** | 1 |

**Abbreviations:** LOS: length of stay; CC: comorbidity and complication; **: *P* < 0.01; *: *P* < 0.05.
